# Utilizing numerical simulations to prevent stent graft kinking during thoracic endovascular aortic repair

**DOI:** 10.1016/j.jvscit.2023.101269

**Published:** 2023-07-17

**Authors:** Tim J. Mandigers, Anna Ramella, Daniele Bissacco, Maurizio Domanin, Joost A. van Herwaarden, Giulia Luraghi, Francesco Migliavacca, Santi Trimarchi

**Affiliations:** a Section of Vascular Surgery, Cardio Thoracic Vascular Department, Fondazione IRCCS Cà Granda Ospedale Maggiore Policlinico, Milan, Italy; b Department of Vascular Surgery, University Medical Center Utrecht, Utrecht, The Netherlands; c Department of Chemistry, Materials and Chemical Engineering “G. Natta”, Politecnico di Milano, Milan, Italy; d Department of Clinical Sciences and Community Health, Università degli Studi di Milano, Milan, Italy

**Keywords:** Computational modeling, Finite element analysis, Patient-specific in silico model, TEVAR, Thoracic endovascular aortic repair

## Abstract

Numerical simulations of thoracic endovascular aortic repair (TEVAR) may be implemented in the preoperative workflow if credible and reliable. We present the application of a TEVAR simulation methodology to an 82-year-old woman with a penetrating atherosclerotic ulcer in the left hemiarch, that underwent a left common carotid artery to left subclavian artery bypass and consequent TEVAR in zone 2. During the intervention, kinking of the distal thoracic stent graft occurred and the simulation was able to reproduce this event. This report highlights the potential and reliability of TEVAR simulations to predict perioperative adverse events and short-term postoperative technical results.

Numerical simulations that virtually reproduce thoracic endovascular aortic repair (TEVAR) represent innovative computational adjuncts that may potentially aid the preprocedural planning phase in the future by predicting perioperative or short-term postoperative technical events and results.^1^ Such tools could be further optimized regarding their credibility and reliability by providing evidence of their effectiveness and workflow, as illustrated in this study that applied a recently developed TEVAR simulation methodology to a patient-specific case with preoperative distal thoracic aortic stent graft kinking. The patient provided informed consent for the publication of this case report and related imaging.

## Case Report

**Fig 1:**
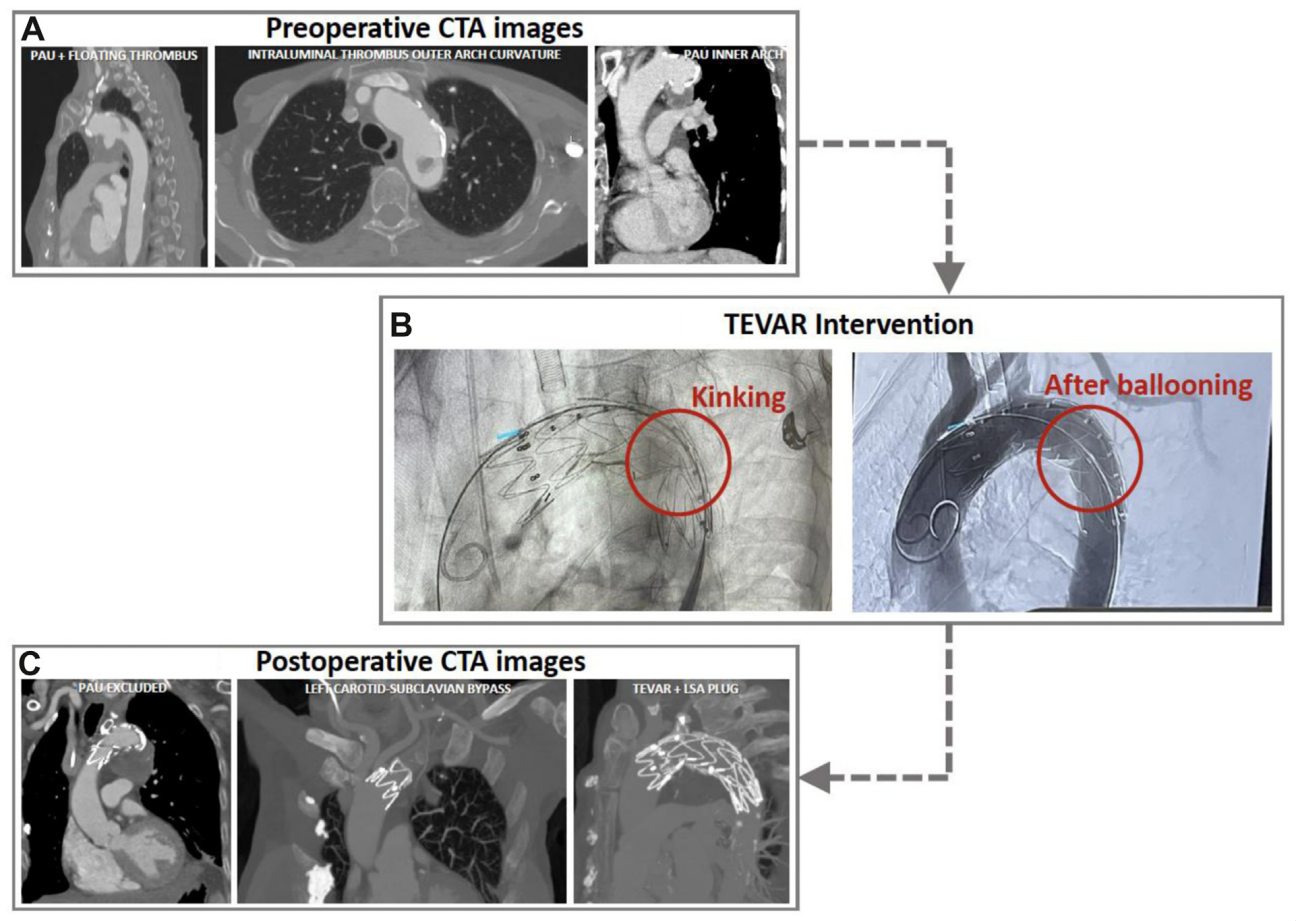
**(A)** Preoperative computed tomography angiography (*CTA*) imaging. **(B)** Fluoroscopy illustrating the distal stent graft kinking, resolved by ballooning as shown on the final angiogram. **(C)** Technical results as seen on postoperative CTA after 8 days. *LSA*, left subclavian artery; *PAU*, penetrating atherosclerotic ulcer; *TEVAR*, thoracic endovascular aortic repair.

An 82-year-old woman with hypertension and a history of heavy smoking (approximately 20 cigarettes per day) presented with a penetrating atherosclerotic ulcer (PAU) in the left hemiarch with maximum axial diameters of 38 × 37 mm on computed tomography angiography (CTA). There were no further relevant cardiovascular diseases or interventions in her medical history. Additionally, an intraluminal floating thrombus located at the outer curvature of the proximal descending aorta (approximately 18 mm length, 15 × 8 mm diameter) was identified on CTA, that seemed to be connected to the intraluminal thrombus of the PAU anteriorly (Fig 1, *A*).

First, a left common carotid artery (LCCA) to left subclavian artery (LSA) bypass was performed, to obtain an adequate proximal landing zone in zone 2 for TEVAR. During the same intervention, a Valiant thoracic aortic stent graft with the Captivia delivery system (Valiant Captivia, VAMF3232C100TU) (Medtronic Inc., Minneapolis, MN) was deployed followed by a plug at the LSA origin to prevent retrograde type II endoleak. Interestingly, during the intervention, a kinking of the thoracic stent graft occurred between the fifth and sixth nitinol stent rings at the distal portion of the thoracic stent graft in zone 4, which was resolved after ballooning this portion of the stent graft (Fig 1, *B*).

The postoperative course was regular, without any adverse events, including neurological and peripheral thromboembolic. Postoperative CTA after 8 days showed adequate exclusion of the PAU and intraluminal thrombus, without endoleak and patency of the Valiant Captivia and LCCA to LSA bypass (Fig 1, *C*). Discharge was on postoperative day 11. During follow-up, color Doppler ultrasound examination showed adequate patency and flow over the LCCA-LSA bypass. No further follow-up diagnostic imaging has been performed to date.

**Fig 2:**
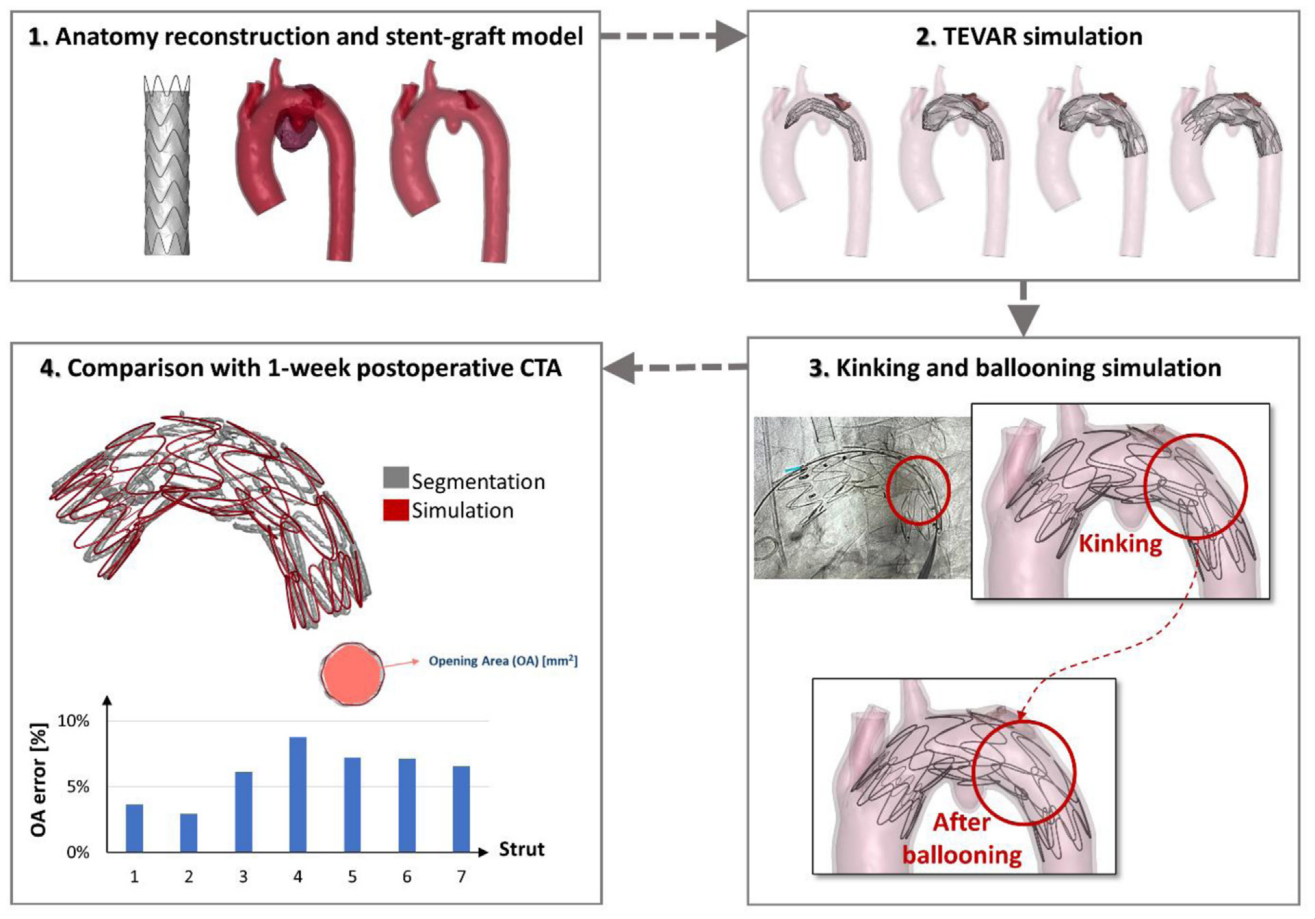
Workflow of the numerical simulation and comparison of the simulation results with postoperative computed tomography angiography (*CTA*) image segmentation. *TEVAR*, thoracic endovascular aortic repair.

The patient-specific ascending, arch, and descending aortic anatomy were reconstructed from preoperative CTA images, including the intraluminal thrombus in zone 3. A recently developed high-fidelity numerical methodology^2^^,^^3^ was adopted to simulate Valiant Captivia deployment in the reconstructed patient-specific anatomy. Simulations were carried out using the commercial finite element LsDyna software (Ansys Inc., Canonsburg, PA) on 28 CPUs and 250 GB of RAM memory. The device model incorporated nitinol stent prestress and underwent complete mechanical characterization.^2^ As during the intervention, a Valiant Captivia was deployed at the distal border of the LCCA. The numerical method was able to reproduce the kinking of the thoracic stent graft between the fifth and sixth nitinol rings. Ballooning of the stent graft was virtually replicated as well, to resolve the kinking (Fig 2, Supplementary Video, online only). The reliability of the simulation was evaluated by qualitatively comparing the stent graft configuration segmented from postoperative CTA images with the numerical results obtained by the simulation: there was a satisfactory overlap (Fig 2). In terms of quantitative assessment, the opening area at each nitinol stent ring (expressed as a percentage error between the simulation and CTA segmentation in square millimeters) remained <10%, with higher values in the region of the thrombus at the outer arch curvature (Fig 2).

## Discussion

**Fig 3:**
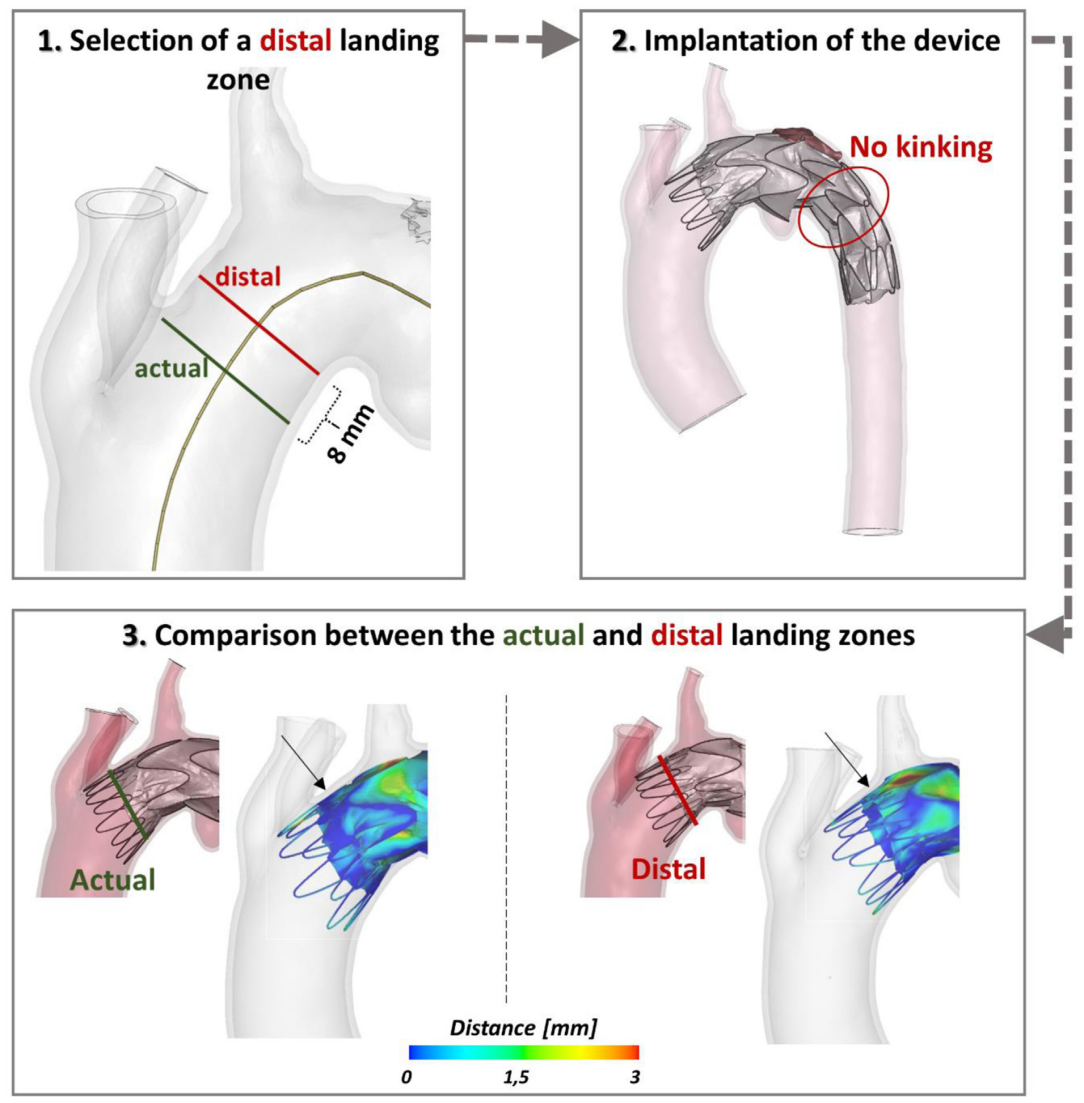
Virtual scenario of a more distal landing zone and comparison to the actual one in terms of distance between the stent graft and the aortic wall.

The potential impact of applying this methodological TEVAR simulation model with Valiant Captivia to patient-specific cases could be of significant value during preprocedural planning given the ability to predict both perioperative adverse events (such as kinking for the illustrated case) and short-term postoperative technical results. As an additional tool, it may serve physicians in choosing the optimal proximal and/or distal sealing zones in specific cases with challenging aortic anatomy. In fact, as depicted in Fig 3, a simulation was performed to evaluate the stent graft apposition and kinking with a more distal landing zone. In this scenario, we noted that the distal kinking disappeared and that the third nitinol stent ring bulged into the PAU. However, this configuration might not be optimal, not only because of proximal landing zone reduction, but also because of the increased distance between the aorta and stent graft.

The time to obtain virtual results is compatible with the time needed to plan elective TEVAR; this procedure can be performed in 1 day. Furthermore, other patient-specific components such as the presence of intraluminal thrombus (as for this case) or calcifications, can be included in the simulation, to evaluate their impact on the stent graft deployment. Virtual deployment of different thoracic aortic stent grafts of different manufacturers, if verified and validated,^4^ may also help to find the most suitable device with optimal sealing for different patient-specific aortic anatomies that vary according to geometrical characteristics (eg, diameter, length, angulation, and tortuosity).

Similar to the present case, another illustrative case by Derycke et al^5^ has previously demonstrated the potential of a FE custom-made double branch Relay (Terumo Aortic, Sunrise, FL) TEVAR simulation for an aortic arch aneurysm, to reliably find stent graft collapse that led to postoperative complications. This TEVAR simulation found the deformation of the three nitinol stent rings at the same location as seen on postoperative CTA. Also in our case, the simulation was performed after the clinical procedure to verify if the numerical model was able to predict the perioperative stent graft kinking during the intervention. In our patient-specific case, the event was managed promptly by using a balloon without clinical and technical consequences.

## Conclusions

This study further highlights the potential and reliability of TEVAR simulations to be adopted in and facilitate preprocedural planning in the future. For example, they could investigate optimal proximal landing and stent graft apposition or the ideal stent graft model in demanding aortic anatomies. One of the challenges before a wider implementation of such tools in daily clinical cardiovascular practice, remains the need to further enhance simulations regarding their reliability and credibility, by providing evidence of their effectiveness and workflow, as illustrated by this case.

## Appendix (online only)

Supplementary Video (online only) Different deployment steps of the virtual thoracic endovascular aortic repair (TEVAR) procedure.

## References

[1] Mandigers T.J., Ramella A., Bissacco D. (2023). Thoracic stent graft numerical models to virtually simulate thoracic endovascular aortic repair: a scoping review. Eur J Vasc Endovasc Surg.

[2] Ramella A., Migliavacca F., Rodriguez Matas J.F. (2022). Validation and verification of high-fidelity simulations of thoracic stent-graft implantation. Ann Biomed Eng.

[3] Ramella A., Migliavacca F., Rodriguez Matas J.F. (2023). Applicability assessment for in-silico patient-specific TEVAR procedures. J Biomech.

[4] American Society of Mechanical Engineers (2018).

[5] Derycke L., Avril S., Perrin D., Albertini J.N., Cochennec F. (2022). Computer simulation model may prevent thoracic stent-graft collapse complication. Circ Cardiovasc Imaging.

